# Sustainable diets reduce diet-related greenhouse gas emissions and improve diet quality: results from the MyPlanetDiet randomized controlled trial

**DOI:** 10.1016/j.ajcnut.2025.05.015

**Published:** 2025-08-26

**Authors:** Katie P Davies, Eileen R Gibney, Ursula M Leonard, Leona Lindberg, Jayne V Woodside, Mairead E Kiely, Anne P Nugent, Elena Arranz, Marie C Conway, Sinead N McCarthy, Aifric M O’Sullivan

**Affiliations:** 1 Institute of Food and Health, School of Agriculture and Food Science, University College Dublin, Dublin, Ireland; 2 Cork Centre for Vitamin D and Nutrition Research, School of Food and Nutritional Sciences, University College Cork, Cork, Ireland; 3 Centre for Public Health, School of Medicine, Dentistry and Biomedical Sciences, Queen’s University Belfast, Belfast, Northern Ireland, United Kingdom; 4 Institute for Global Food Security, School of Biological Sciences, Queen’s University Belfast, Belfast, United Kingdom; 5 Instituto de Investigación en Ciencias de la Alimentación, CIAL (CSIC-UAM, CEI UAM+CSIC), Universidad Autónoma de Madrid, Madrid, Spain; 6 Department of Agrifood Business and Spatial Analysis, Teagasc Food Research Centre, Ashtown, Dublin, Ireland

**Keywords:** diet-related greenhouse gas emissions, sustainable diet, personalized nutrition, dietary intervention, food-based dietary guidelines

## Abstract

**Background:**

Diet-related environmental impacts must be reduced to mitigate climate change. Although many sustainable diets have been proposed, the human and planetary impacts of following such diets have not been tested.

**Objective:**

The aim of this study was to assess health and environmental outcomes related to following whole-diet sustainable dietary advice.

**Methods:**

The MyPlanetDiet RCT was a 12-week single-blinded, parallel study testing the impacts of a more sustainable diet. Participants were randomly assigned to receive personalized advice based on a sustainable diet (intervention) or based on current healthy eating guidelines (control). Dietary assessments, fasted anthropometry, and fasted serum samples were collected at baseline and end point. The primary outcome was change in diet-related greenhouse gas emissions (GHGE) measured in kilograms carbon dioxide equivalents per day (kgCO_2_-eq/d). Secondary outcomes included changes in diet quality, macronutrient and food group intakes, diet-related water footprint, and health biomarkers. Data were analyzed using 2-way mixed analysis of covariance.

**Results:**

Study participants (*n* = 292) decreased diet-related GHGE over time (*P* < 0.001) with a significant time × group interaction between control (from 6.5 ± 0.2 to 5.7 ± 0.2 kgCO_2_-eq/d) and intervention groups (7.1 ± 0.2 to 4.8 ± 0.1 kgCO_2_-eq/d; *P* < 0.001). Diet quality increased in control (from 44.2 ± 0.8 to 52.9 ± 0.9) and intervention (from 44.7 ± 0.8 to 53.0 ± 0.9) groups (*P* < 0.001). Participants decreased red meat intakes (control: from 34.2 ± 2.9 to 25.7 ± 2.4 g/d; intervention: from 42.7 ± 3.4 to 12.8 ± 1.9 g/d; *P* < 0.001) and increased plant-based food intakes including beans, peas, and lentils (control: from 15.4 ± 1.9 to 18.3 ± 2.1 g/d; intervention: from 18.4 ± 2.1 to 49.2 ± 4.3 g/d; *P* < 0.001), fruit (control: from 164.8 ± 12.3 to 264.5 ± 13.9 g/d; intervention: from 188.5 ± 14.2 to 233.7 ± 13.5 g/d; *P* < 0.001), and vegetables (control: from 148.1 ± 6.5 to 163.1 ± 7.3 g/d; intervention: from 161.3 ± 5.9 to 201.9 ± 8.0 g/d; *P* < 0.001). No changes in anthropometry, serum biochemistry, or diet-related water footprint were observed.

**Conclusions:**

Personalized sustainable dietary advice led to healthier diets and lower diet-related GHGE with no short-term negative health effects.

This trial was registered with clinicaltrials.gov as NCT05253547 (https://clinicaltrials.gov/study/NCT05253547).

## Introduction

Human activities, including agriculture and food production, contribute to global warming and climate change [1]. Many countries have pledged to reduce environmental impacts, including greenhouse gas emissions (GHGEs), leading to mounting pressure to decarbonize all human activities [2]. However, unlike other sectors, agriculture and food production cannot be decarbonized quickly [3]. Producing food releases GHGE, with some foods giving rise to more GHGE than others [4]. In general, animal-based foods, ruminant meat in particular, give rise to more GHGE than plant-based foods [4]. Modeling analyses show the greatest potential to reduce diet-related GHGE is to remove animal-based foods from the diet [5]. However, when moving toward a lower greenhouse gas (GHG)-emitting diet or a more sustainable diet, one must also consider what is healthy, nutritionally adequate, accessible, and acceptable [6]. Sustainable diets are high in plant-based foods like whole grains, fruits, vegetables, and plant-based proteins with low-to-moderate intakes of animal-based foods [6,7]. Such recommendations are similar to the advice in food-based dietary guidelines (FBDGs) [8]. However, adherence to FBDGs remains low [[9], [10], [11]]. For example, <0.1% of participants in the United Kingdom who took part in the National Diet and Nutrition Survey met all 9 UK FBDG recommendations [10]. Thus, significant dietary behavior change is needed to improve adherence to FBDGs [8], and further dietary change beyond FBDG will be needed to meet climate targets [12]. To achieve this, a whole-diet approach to reduce diet-related environmental impacts and improve dietary intakes is needed [10,13,14]. Most studies describing the impacts of whole-diet shifts toward sustainability are based on modeling or observational data, with no direct evidence that people can adopt such diets in practice [5,7].

There are concerns and potential risks associated with more sustainable dietary patterns, including risk of not meeting nutrient requirements for all [13,14]. At the same time, poor consumer awareness and acceptability present barriers to adopting a more sustainable diet [15,16]. It is therefore important to consider alternative approaches for delivering dietary change. In previous studies, recommendations personalized to an individual led to greater and longer-lasting dietary change than a 1-size-fits-all approach [17,18]. Personalized nutrition provides tailored feedback based on a variety of individual characteristics, including nutrient requirements, nutrition-related phenotypes, and genetics [19]. Standardized methods of providing such dietary advice, like decision trees, have been developed [20]. Studies using decision trees show that personalized nutrition can be scalable, reproducible, and effective in improving diet quality [17]. As considerable dietary change will be needed to achieve healthier and more sustainable diets, these methods may support this transition. Understanding outcomes of sustainable dietary advice can help inform future advice on sustainable diets. However, there is a lack of human intervention studies examining impact and efficacy of recommendations for sustainable diets. To our knowledge, the MyPlanetDiet study is the first RCT to test a whole-diet approach for sustainable diets [21], with a primary aim to test the impact of following a sustainable diet on diet-related GHGE. Secondary outcomes include changes in food group intakes, nutrient intake, metabolic health indicators (anthropometry and clinical chemistry biomarkers), and diet-related water footprint.

## Methods

### Study design

MyPlanetDiet was a parallel RCT with 2 groups (intervention and control). The intervention group received personalized nutrition feedback for a sustainable healthy diet. The control group received personalized nutrition feedback based on existing healthy eating guidelines (HEGs) on the island of Ireland [22]. Participants were randomly assigned to either control or intervention group before their first study visit (baseline) and were asked to follow the personalized dietary advice for 12 weeks (end point). The primary outcome of the MyPlanetDiet study was a reduction in diet-related GHGE. Other study outcomes included changes in dietary intake (food groups, macronutrients, and diet quality), anthropometry, serum biochemistry, and diet-related water footprint. The full MyPlanetDiet methodology has been reported elsewhere [21].

### Study participants

MyPlanetDiet recruited healthy adults aged 18–64 years in areas surrounding the 3 study sites: University College Dublin, University College Cork, and Queen’s University Belfast. The Human Research Ethics Committee in University College Dublin (LS-21-51-Davies-OSullivan) and the Clinical Research Ethics Committee of the Cork Teaching Hospitals in University College Cork [ECM 4 (cc) 10/8/2021 and ECM 3 (f) 19/10/2021] granted ethical approval for the study. The Faculty of Medicine, Health and Life Sciences Research Ethics Committee, Queen’s University Belfast affirmed the ethical approval granted in University College Dublin (MHLS_21_109). The study abided by the tenets of the Declaration of Helsinki. MyPlanetDiet was registered with clinicaltrials.gov (NCT05253547). Study researchers developed a centralized standard operating procedure (SOP) for managing and conducting the trial.

The study aimed to recruit 360 participants (*n* = 120 at each site). Inclusion criteria included general good health, free of any conditions that may impact study outcomes, and following a moderate-to-high GHG-emitting diet. Moderate-to-high diet-related GHGE was determined by having higher red meat intake than the mean daily average in the general population or self-reported red meat intake of 3 or more servings per week [23]. Exclusion criteria included those who were currently pregnant or lactating, following a medically prescribed diet, food allergies affecting study adherence, being immunocompromised, excessive alcohol intake (≥28 standard units per week), regular consumption of single high-dose vitamin or mineral supplements, participation in another research study, or inability to read or write in English. Following written informed consent, participants were stratified by sex and age group and randomly assigned using site-specific blocked randomization lists. Participants were blinded to their group, however, due to the nature of the dietary advice provided, researchers were not able to be blinded.

### Dietary assessment

Participants completed dietary assessments and a health and lifestyle questionnaire before attending their first onsite visit (baseline). The health and lifestyle questionnaire included sociodemographic factors (sex, age, race, education, living location, household size, and employment status) and lifestyle factors (smoking use, alcohol use, and physical activity). Dietary assessments were repeated at the end of the 12-wk intervention (end point). Each dietary assessment included 3 online 24-h dietary recalls using Foodbook24 [24]. Foodbook24 was validated in a similar cohort against a 4-d weighed food diary and nutrient status biomarkers [24]. The Foodbook24 methodology and the development of the food list have been previously described [24,25]. Within this study, participants completed 24-h recalls on 3 nonconsecutive days and were asked to recall food and drinks consumed the previous day. All reported foods consumed were grouped into food groups for personalized feedback. Single food items were directly categorized into the appropriate food group (eg, apples grouped as fruit). Mixed dishes were disaggregated using a recipe database, as previously described, where foods within the mixed dishes were identified, quantified, and then categorized into the appropriate food group [21]. The mean daily weight in grams for each food group was calculated and used to provide the personalized feedback for study participants. Dietary data from these 24-h recalls were used to measure mean daily macronutrient and food group intakes. Micronutrient intakes will be reported separately in an additional manuscript. Mean daily dietary recall data were also used to calculate Healthy Eating Index scores, as previously described [26].

Environmental impact factors were assigned at a food-level for all reported intakes [21]. Environmental data were used from previously published work by Colombo et al. [27], selected due to proximity of the data (United Kingdom) to the island of Ireland and for the methodological consideration of factoring the volume of food eaten that is produced locally compared with those imported to the United Kingdom [27]. Individual foods were mapped with corresponding environmental metrics where possible. For mixed dishes, the recipe database described earlier was used to calculate environmental metrics by assigning individual ingredients the respective environmental data.

### Personalized feedback

The development and testing of the MyPlanetDiet personalized nutrition feedback has been previously described [21]. Briefly, the control participants received feedback for a healthy diet using a personalized approach based on existing HEGs, while the intervention group received personalized feedback for a more sustainable and healthy diet (Table 1). Researchers generated personalized feedback for each participant by analyzing baseline dietary intake and using a developed network of decision trees and feedback messages to create a personalized report to discuss with the participants in a 1-to-1 diet consultation session at their baseline visit. Feedback messages included recommendations for target intakes of food groups (grams per day or grams per week) and directional guidance (increase, decrease, or maintain). Personalized nutrition feedback was tailored to an individual’s dietary intake and nutritional requirements (eg, age, sex, and energy requirements). Control group participants received feedback on 5 food groups based on the order they are presented in the HEGs (Table 1). The intervention group participants received personalized advice for 6 food groups in the order presented in Table 1. Participants were not prescribed specific foods within these food groups. Specific differences between the feedback provided to intervention and control groups are as follows. The intervention group was the only group to receive feedback on intakes of plant proteins including legumes, soy products, meat alternatives, nuts, and seeds. Although both groups received feedback on fruit and vegetable intakes, only the control group included messaging on fruit juice and citrus fruits (Table 1). The intervention group received feedback on intakes of dark green vegetables and red/orange vegetables. Some food groups were included in both control and intervention feedback but the content and target for messaging differed. For example, red meat intake in the control group was defined as pork, beef, or lamb and limited to 70 g/d. The intervention group recommendation included no >1 serving of 140 g of red meat per week where red meat was defined as beef and lamb products (Table 1). Further details on the personalized nutrition feedback are given in our protocol article [21]. Feedback reports also included a visual representation of nutrient intakes compared with European Food Safety Authority daily reference values using a traffic light system, previously described in Food4Me [20,28]. All participants were given their personalized report and resource booklets with some nutrition information and generic advice to take home and were asked to follow their dietary advice for 12 wk. All participants received check-in calls or e-mails at weeks 1, 3, 6 and 9. Following the study period, the number of feedback messages each participant received asking them to change intakes of a particular food group was quantified and assessed alongside dietary changes.

**TABLE 1:** Food groups included in personalized nutrition feedback for the control and intervention groups and range of recommended intake.

Control group recommendations		Intervention group recommendations	
Food group	Recommendation (g/d)	Food group	Recommendation (g/d)
Fruit and vegetables		Meat	
Total fruit and vegetables	400	Red meat	0–20
Citrus fruit	80	Poultry, pork and eggs	0–65
Juice	0–125	Plant protein	
Total vegetables	240	Legumes	125–150
Whole grain		Nuts and seeds	25–35
Total whole grain	≥50	Soy products and meat alternatives	0–42
Dairy		Dairy	
Servings of dairy	3 servings	Total dairy equivalents	200–300
Type of dairy (whole, low-fat, skimmed, etc).	All low-fat or skimmed	Cheese	0–25
Fish		Fish	
Total fish	28	Total fish	28
Oily fish	14	Oily fish	14
Red and processed meat		Fruit and vegetables	
Processed meat	0	Total fruit	200–300
Lean red meat	≤70	Total vegetables	300
Protein servings per d	2 servings	Dark green vegetables	100
		Red/orange vegetables	100
		Starches	
		Total whole grains	≥50
		Starchy vegetables	0–150
		Total servings of starches	4–7 servings

Food groups in order as presented to the participant. Further details in Davies et al. [21].

### Clinical measurements

Fasting clinical measurements including height, body composition, waist circumference, and blood pressure were recorded at baseline and end point. Freestanding Leicester stadiometers (Seca) were used to measure height, and bioimpedance body composition analyzers (BC-420MA; Tanita) were used to measure weight and body composition. Blood pressure monitors (Omron Healthcare) were used to measure blood pressure, with participants rested and seated, with feet on the ground and arm resting on a flat surface.

### Biological sample collection and analysis

Trained phlebotomists collected fasting serum samples (BD evacuated tube) from participants. Serum samples were processed according to the study SOP [21]. Metabolic health markers were measured in the Mater Misericordiae University Hospital (MMUH; Dublin, Ireland), an Irish National Accreditation Board laboratory that operates in compliance with International Standard ISO 15189 and the European Union blood Directive 2002/98/EC. Samples were analyzed according to MMUH SOP using the Alinity c Clinical Chemistry Analyser (Abbott Laboratories) and corresponding reagent kits. Quality and accuracy were monitored using internal quality controls in line with manufacturer’s guidelines. Total cholesterol, LDL cholesterol, HDL cholesterol, triglycerides, insulin, glucose, and C-reactive protein were measured using serum aliquots. Per MMUH SOP, where triglyceride concentration was <2.2 mmol/L, LDL cholesterol was calculated using Friedewald equation. When triglyceride concentrations were >2.2 mmol/L, the directly measured LDL cholesterol concentration value was used. When C-reactive protein concentration was below the lowest level of detection (0.4 mmol/L), the median between zero and the lowest level of detection was assigned.

### Statistical analysis

Sample size was calculated based on a 20% difference in diet-related GHGE between control and intervention groups at end point (80% power; 5% significance). The required sample size was 360 participants in total (120 per site), allowing for a 25% dropout rate. Therefore, the minimum needed to complete the study was 270 participants. Statistical analysis was completed using IBM SPSS Statistics for Macintosh, version 29.0. Demographic data are presented as count (*n*) and percentage or mean ± SE. Histograms and Shapiro-Wilk tests were used to test for normal distribution for continuous variables. Variables that were not normally distributed were transformed using logarithmic or square root functions. Two-way mixed analysis of covariance compared changes in diet-related GHGE, food group and macronutrient intakes, diet-related water footprint, and clinical measurements. Pearson correlation was used to identify covariates. Sex (male/female), age (years), BMI (in kg/m^2^), and energy intake (kilocalories) were considered as covariates for analysis. *P* values of <0.05 were considered statistically significant. Bonferroni post hoc tests were used to assess change over time within group for the primary outcome (diet-related GHGE). Analysis was repeated for those with energy intakes of >1.1× resting metabolic rate, assessed via the Mifflin–St Jeor equation [29]. The results were similar to the full sample population and can be found in Supplemental Tables 1–3.

## Results

**FIGURE 1:**
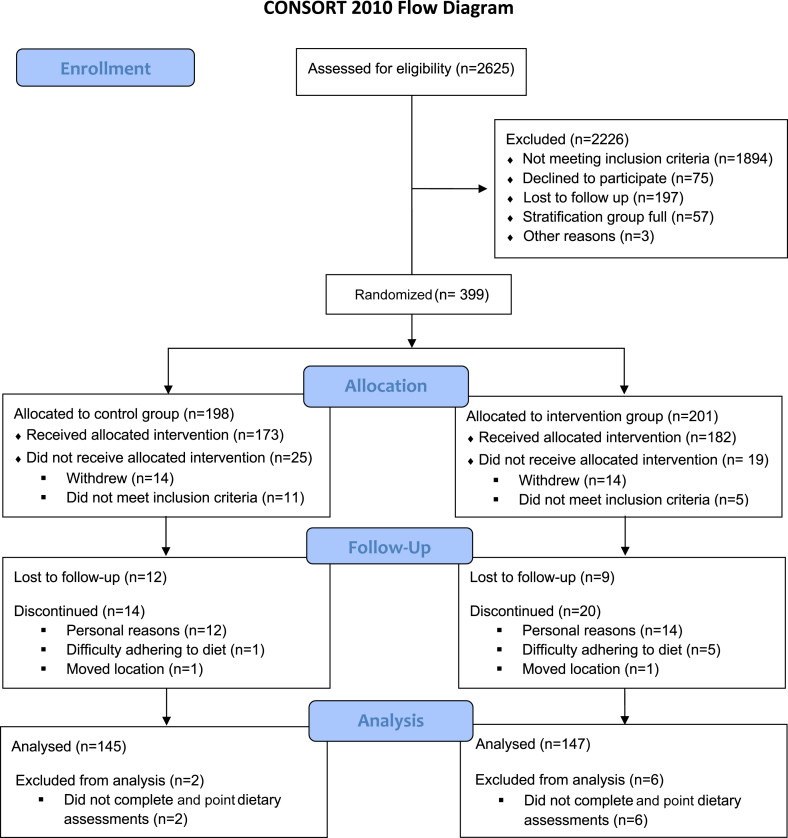
CONSORT Flow Diagram of MyPlanetDiet participants.

There were 2625 responses to online screening questionnaires between February 2022 and March 2023 (Figure 1). Over 84% of individuals who completed screening were excluded, with 72% of respondents not meeting inclusion criteria (Figure 1). A further 3% declined to participate, 9% lost to follow up, and 3% were unable to take part due to full stratification groups. Across the 3 study sites, 399 participants were randomly assigned, and 355 participants began the study. Intervention data were collected between March 2022 and May 2023. During the study, 16% were lost to follow up or discontinued the intervention for personal reasons. Participants who completed 2 or more 24-h dietary recalls at end point were included, resulting in 292 participants in the paired analysis (Figure 1). Baseline demographics are presented in Table 2. The mean age was similar in control (42.3 ± 1.1 y) and intervention (42.2 ± 1.0 y) groups. More females than males took part in the study with no significant differences in sex distribution between the control and intervention groups. The sample was most likely to identify as White (95%), with a third-level degree (73%) and living an urban area (45%).

**TABLE 2:** Demographic characteristics of the MyPlanetDiet cohort, split by intervention group.

	Control (*n* = 145)		Intervention (*n* = 147)	
Age, mean (SE)	42.3	1.1	42.2	1.0
Sex, *n* (%)				
Male	59	40.7	69	46.9
Female	86	59.3	78	53.1
Race, *n* (%)				
White	139	95.9	137	93.2
Other	6	4.1	10	6.8
Living location, *n* (%)				
Open country/village	39	26.9	33	22.4
Small town	22	15.2	25	17.0
Large town	18	12.4	25	17.0
City	66	45.5	64	43.5
Education, *n* (%)				
No third-level degree	39	26.9	39	26.5
Third-level degree	47	32.4	38	25.9
Postgraduate	59	40.7	70	47.6
Household children, *n* (%)				
0	76	52.4	70	47.6
1–2	44	30.4	58	39.5
3–4	25	17.3	19	12.9
Smoking history, *n* (%)				
Never smoker	101	69.7	92	62.6
Past smoker	38	26.2	47	32.0
Current smoker	6	4.1	8	5.4
Alcohol intake (units/wk), *n* (%)				
0	25	17.2	31	21.1
1–11	91	62.8	85	57.8
12–17	19	13.1	16	10.9
≥18	10	6.9	15	10.2
Self-reported physical activity (min/d), mean (SE)				
Vigorous	29.1	2.7	25.3	2.5
Moderate	31.0	2.6	40.1	4.8
Walking	56.4	4.6	56.7	5.5
Estimated energy needs (kcal), mean (SE)	2518.2	45.7	2555.0	44.3

Continuous variables presented as mean (SE). Categorical variables presented as *n* (%). Race was self-reported from the following options: White, Black or African American, South Asian, East Asian, or other (free text). Education was self-reported from the following options: no formal/primary, lower secondary, higher secondary, third-level nondegree, third-level degree, and postgraduate.

### Diet-related environmental impacts

There was a significant effect of time for diet-related GHGEs (*P* < 0.001) with reductions in both the control (6.5 ± 0.2 kg CO_2_-equivalents/d to 5.7 ± 0.2 kg CO_2_ equivalents/d) and intervention groups (7.2 ± 0.3 kg CO_2_-equivalents/d to 4.8 ± 0.2 kg CO_2_ equivalents/d) (Table 3). Greater reductions in the intervention group led to a significant time × group interaction for diet-related GHGE (*P* < 0.001) and diet-related GHGE per 2500 kcal (*P* < 0.001). Post hoc analysis revealed significant differences over time in diet-related GHGE between baseline and end point in the intervention group (*P* = 0.003), but not in the control group (*P* = 0.076). No changes over time or between groups were observed for diet-related water footprint or water footprint per 2500 kcal (Table 3).

**TABLE 3:** Mean daily diet-related environmental impacts at baseline and end point, split by intervention group.

	Control (*n* = 145)				RM ANCOVA, *P*	Intervention (*n* = 147)				RM ANCOVA, *P*	2-way ANCOVA, *P*	
	Baseline		End point			Baseline		End point				
	Mean	SE	Mean	SE		Mean	SE	Mean	SE		Time	Time × Group
GHGE (kg CO_2_-eq)	6.5	0.2	5.7	0.2	0.076	7.2	0.3	4.8	0.2	0.003	<0.001	<0.001
GHGE/2500 kcal	7.9	0.2	7.8	0.2	0.628	8.1	0.2	6.7	7.9	<0.001	<0.001	<0.001
WF (L H_2_O)	766.2	46.3	717.0	46.4	—	790.4	45.2	680.0	38.5	—	0.443	0.308
WF/2500 kcal	962.4	61.0	1010.4	68.2	—	913.5	53.5	967.8	57.9	—	0.119	0.755

Two-way mixed ANCOVA was used to measure significance. Pearson correlation was used to identify covariates. ANCOVA controlled for energy where significantly correlated. *P* value < 0.05 was considered significant. Post hoc analysis within group was performed when significant time × group interaction.

Abbreviations: GHGE (kg CO_2_-eq), greenhouse gas emissions (kilograms carbon dioxide equivalents); RM ANCOVA, repeated measure analysis of covariance; WF (L H_2_O), water footprint (liters of water).

### Food group intakes

Two-thirds of total participants (34% of control and 98% of intervention) received 5 or more personalized feedback messages to change intakes of a particular food group (Supplemental Table 4), with the number and type of feedback messages differing significantly between control and intervention groups (Supplemental Table 5). Overall, significant effects of time were observed, with both control and intervention groups significantly increasing intakes of vegetables (*P* < 0.001), fruit (*P* < 0.001), and fish (*P* < 0.001) (Table 4). Participants in the intervention group increased their intakes of legumes (18.4 ± 2.1 g to 49.2 ± 4.3 g, *P* < 0.001) and nuts and seeds (5.5 ± 0.7 g to 12.9 ± 1.2 g, *P* < 0.001) between baseline and end point and relative to the control group (15.4 ± 1.9 g to 18.3 ± 2.1 g and 5.2 ± 0.7 g to 7.2 ± 0.9 g, respectively). There was a time × group interaction for total dairy intake (*P* < 0.001), driven by the increase in the control group (233.6 ± 13.9 g to 296.1 ± 17.7 g) and decrease in the intervention group (223.5 ± 13.1 g to 172.9 ± 9.3 g). Intervention participants increased intakes (13.0 ± 6.3 g to 47.1 ± 8.4 g), while control participants decreased intakes of dairy alternatives (9.1 ± 3.1 g to 8.1 ± 2.6 g) relative to baseline (*P* < 0.001). Both groups reduced intakes of red meat and poultry, pork, and eggs over time (*P* < 0.001, *P* = 0.005), but there were significant time × group interactions in both red meat (*P* < 0.001) and poultry, pork, and eggs (*P* < 0.001), driven by larger reductions in the intervention group than those in the control group.

**TABLE 4:** Mean daily food group intake at baseline and end point, split by intervention group.

	Control (*n* = 145)				Intervention (*n* = 147)				*P*	
	Baseline		End point		Baseline		End point			
	Mean	SE	Mean	SE	Mean	SE	Mean	SE	Time	Time × group
Total grains (g)	301.0	11.8	292.2	11.9	290.0	9.8	300.3	11.4	0.305	0.241
Whole grains (g)	31.3	2.2	40.8	2.5	27.1	2.1	43.1	2.6	0.276	0.048
Tubers/starchy vegetables (g)	81.2	6.0	83.1	6.1	95.9	5.9	77.4	5.1	0.074	0.051
Vegetables (all) (g)	148.1	6.5	163.1	7.3	161.3	5.9	201.9	8.0	<0.001	0.068
Dark green (g)	31.8	2.7	42.6	3.3	36.8	2.7	55.2	3.4	<0.001	0.202
Red/orange (g)	70.6	3.9	73.6	3.9	73.3	3.4	90.5	5.1	0.015	0.198
Other (g)	45.6	2.9	46.9	2.9	51.2	3.0	56.2	3.4	0.058	0.167
Fruit (g)	164.8	12.3	264.5	13.9	188.5	14.2	233.7	13.5	<0.001	0.009
Dairy (g)	233.6	13.9	296.1	17.7	223.5	13.1	172.9	9.3	0.564	<0.001
Cheese (g)	12.8	1.2	11.7	1.1	15.2	1.4	12.7	1.1	0.666	0.457
Liquid dairy (g)	220.8	14.0	284.4	17.3	208.3	13.0	160.2	9.2	0.626	<0.001
Dairy alternatives (g)	9.1	3.1	8.1	2.6	13.0	6.3	47.1	8.4	<0.001	<0.001
Animal protein (g)										
Red meat (beef/lamb)	34.2	2.9	25.7	2.4	42.7	3.4	12.8	1.9	<0.001	<0.001
Pork, poultry and eggs	142.7	8.9	115.4	6.3	132.9	7.4	67.3	5.0	0.007	<0.001
Fish	17.5	2.1	37.8	3.5	23.8	2.5	32.9	2.9	<0.001	0.060
Plant protein										
Legumes (g)	15.4	1.9	18.3	2.1	18.4	2.1	49.2	4.3	<0.001	<0.001
Soy and meat alternatives (g)	0.9	0.5	3.7	1.7	2.8	1.0	12.0	1.8	<0.001	<0.001
Nuts and seeds (g)	5.2	0.7	7.2	0.9	5.5	0.7	12.9	1.2	0.100	<0.001

Significance was measured with 2-way mixed analysis of covariance (ANCOVA). Pearson correlation was used to identify covariates. ANCOVA controlled for energy where significantly correlated. *P* value of <0.05 was considered significant.

### Nutrient intakes

Energy intakes significantly decreased at end point in both groups (*P* = 0.030), with a significant time × group interaction (*P* = 0.003) driven by a larger difference in the intervention group (2211.6 ± 44.7 kcal to 1807.7 ± 40.6 kcal) than that in the control group (2108.9 ± 58.6 kcal to 1877.2 ± 54.1 kcal) (Table 5). There was a significant effect of time for both groups with increases in percent total energy from carbohydrate (*P* < 0.001) and polyunsaturated fat (*P* < 0.001). Both groups significantly decreased their percent total energy from fat (*P* < 0.001), saturated fat (*P* < 0.001), and monounsaturated fats (*P* < 0.001). There were significant increases in fiber intakes (*P* < 0.001), with bigger increase in the intervention group (19.1 ± 0.5 g to 22.3 ± 0.7 g) than that in the control group (18.5 ± 0.6 g to 19.7 ± 0.6 g; *P* = 0.020). There was a significant effect of time (*P* < 0.001) and time × group interaction (*P* < 0.001) for protein intake driven by reductions in the intervention group (91.5 ± 2.3 g to 73.6 ± 1.8 g) compared with relatively stable intakes in the control group (89.4 ± 2.6 g to 87.0 ± 2.5 g). There was no significant effect of time for intakes of animal-sourced protein due to relatively stable intakes in the control group. However, there was a significant time × group interaction for intakes of animal-sourced protein (*P* < 0.001) related to greater reductions in the intervention group (64.0 ± 2.0 to 40.6 ± 1.5 g) than those in the control group (63.0 ± 2.2 g to 60.3 ± 1.9 g). There was a significant time × group interaction (*P* = 0.001) for plant-based protein intake driven by an increase in the intervention group (27.5 ± 0.9 g/d to 33.0 ± 1.1 g/d) compared with that in the control group (26.4 ± 1.0 g/d to 26.6 ± 1.0 g/d).

**TABLE 5:** Mean daily nutrient intakes at baseline and end point, split by intervention group.

	Control (*n* = 145)				Intervention (*n* = 147)				*P*	
	Baseline		End point		Baseline		End point			
	Mean	SE	Mean	SE	Mean	SE	Mean	SE	Time	Time × group
Energy (kcal)	2108.9	58.5	1877.2	54.1	2211.6	44.7	1807.7	40.6	0.030	0.003
HEI	44.2	0.8	52.9	0.9	44.7	0.8	53.0	0.9	<0.001	0.792
Carbohydrate (g)	231.9	7.2	218.1	5.9	247.1	5.3	218.3	5.1	<0.001	0.048
Carbohydrate (%)	44.0	0.7	47.0	0.6	44.9	0.5	48.7	0.5	<0.001	0.326
Fiber (g)	18.5	0.6	19.7	0.6	19.1	0.5	22.3	0.7	<0.001	0.020
Fat (g)	87.7	3.1	72.1	2.8	91.2	2.3	71.2	2.1	<0.001	0.361
Fat (%)	37.1	0.6	33.9	0.5	36.8	0.4	34.9	0.5	<0.001	0.157
Saturated fat (g)	34.7	1.5	27.1	1.3	35.8	1.1	24.4	0.9	<0.001	0.024
Saturated fat (%)	14.5	0.3	12.6	0.3	14.3	0.3	11.9	0.2	<0.001	0.151
PUFA (g)	12.4	0.4	11.5	0.4	13.3	0.4	12.9	0.5	0.004	0.294
PUFA (%)	5.3	0.1	5.5	0.1	5.4	0.1	6.3	0.1	<0.001	<0.001
MUFA (g)	31.6	1.1	24.9	1.0	32.5	0.9	25.0	0.8	<0.001	0.726
MUFA (%)	13.4	0.3	11.7	0.2	13.1	0.2	12.3	0.2	<0.001	0.073
Protein (g)	89.4	2.6	87.0	2.5	91.5	2.3	73.6	1.8	<0.001	<0.001
Animal-sourced protein (g)	63.0	2.2	60.3	1.9	64.0	2.0	40.6	1.5	0.135	<0.001
Plant-sourced protein (g)	26.4	1.0	26.6	1.0	27.5	0.9	33.0	1.1	<0.001	0.001
Protein (%)	17.3	0.3	18.9	0.3	16.7	0.3	16.5	0.3	<0.001	<0.001
Protein (g/kg)	1.2	0.0	1.1	0.0	1.2	0.0	1.0	0.0	<0.001	<0.001

Animal-sourced protein included red meat, poultry pork, eggs, fish, and dairy (milk, cheese, and yogurt). Plant-sourced protein included all other food groups (eg, vegetables, grains, nuts, seeds, legumes, meat, and dairy alternatives. Significance measured with 2-way mixed ANCOVA. Pearson correlation used to identify covariates. ANCOVA controlled for energy and/or sex where significantly correlated. *P* value of <0.05 was considered significant.

Abbreviations: ANCOVA, analysis of covariance; HEI, Healthy Eating Index.

### Metabolic health

In total, 273 participants who provided blood samples at baseline and end point (Table 6). There were no significant time or time × group effects on anthropometric measurements or markers of metabolic health (Table 6). Moreover, no clinical differences were observed in blood lipid or metabolic biomarkers during the study (Table 6).

**TABLE 6:** Clinical measurements at baseline and end point, split by intervention group.

	Control (*n* = 141)				Intervention (*n* = 143)				*P*	
	Baseline		End point		Baseline		End point			
	Mean	SE	Mean	SE	Mean	SE	Mean	SE	Time	Time × group
Weight (kg)	80.3	1.6	79.9	1.5	81.7	1.5	81.1	1.5	0.087	0.450
BMI (kg/m^2^)	27.4	0.4	27.2	0.4	27.6	0.4	27.4	0.4	0.497	0.390
Fat mass (kg)	26.4	1.0	26.5	1.0	26.4	1.0	26.0	1.1	0.337	0.076
Muscle mass (kg)	52.0	1.0	51.4	1.0	53.3	0.9	53.0	0.9	0.202	0.113
Waist (cm)	90.1	1.3	89.8	1.3	91.6	1.2	90.3	0.9	0.082	0.086
Hip (cm)	105.9	0.8	105.4	0.8	107.0	0.9	106.3	0.9	0.130	0.628
SBP (mm Hg)	119.4	1.2	118.1	1.2	120.4	1.1	119.8	0.8	0.942	0.594
DBP (mm Hg)	78.4	0.8	78.8	0.8	79.7	0.7	79.6	0.7	0.649	0.472
	Control (*n* = 137)				Intervention (*n* = 136)					
Total-C (mmol/L)	5.26	0.1	5.18	0.1	5.25	0.1	5.16	0.1	0.361	0.506
HDL-C (mmol/L)	1.55	0.0	1.53	0.0	1.51	0.0	1.51	0.0	0.312	0.945
LDL-C (mmol/L)	3.27	0.1	3.19	0.1	3.23	0.1	3.18	0.1	0.084	0.358
TAG (mmol/L)	1.04	0.1	1.04	0.1	1.10	0.1	1.07	0.1	0.753	0.542
Glucose (mmol/L)	5.24	0.1	5.23	0.1	5.30	0.1	5.21	0.1	0.507	0.562
Insulin (pmol/L)	50.6	2.9	51.1	2.8	53.3	3.5	50.9	3.2	0.736	0.823
CRP (mg/L)	1.94	0.2	2.01	0.3	1.87	0.2	1.66	0.2	0.440	0.395

Paired fasting anthropometry was collected from 292 participants (control *n* = 144; intervention *n* = 148) at baseline and end point. Two-way mixed ANCOVA was used to measure significance. Pearson correlation was used to identify covariates. ANCOVA covariates included energy intake, sex, age (years), and/or BMI where significantly correlated. *P* value of <0.05 was considered significant.

Abbreviations: ANCOVA, analysis of covariance; CRP, C-reactive protein; TAG, triglyceride.

## Discussion

To our knowledge, this is the first large multicenter dietary intervention study to show that whole-diet sustainable dietary advice led to significant reductions in diet-related GHGE and improved diet quality without short-term negative health effects. Calls from researchers and public bodies highlight the need to develop strategies to support transition to sustainable healthy diets [7,30]. However, a lack of evidence has slowed incorporating sustainability into healthy eating advice, such as FBDGs. Our results offer new insights on how sustainable dietary advice is interpreted by the public and what dietary changes are made as a result. The study demonstrated that participants in the intervention group were able to diversify their protein intake and introduce more plant-based proteins into their diets. Furthermore, the dietary changes corresponded to the personalized nutrition feedback provided to participants, showing that it is possible to transition to a more sustainable and healthy diet when individuals are appropriately supported.

Until now, evidence for potential environmental impact from dietary changes relied on observational data. A systematic review of 210 diet modeling scenarios found that sustainable diets reduce GHGE by −22% [5]. Five studies modeled partial meat and dairy replacement with plant-based foods, showing a median 31% GHGE reduction [5]. The MyPlanetDiet intervention group had similar reductions in GHGE (33%) with moderate decreases in red meat, poultry, pork, and dairy and increases in plant-based proteins, fruits, and vegetables. Although the greatest potential to reduce diet-related GHGE according to Aleksandrowicz et al. [5] would be following a vegan diet, this type of advice may raise acceptability concerns and could increase risk of certain nutrient deficiencies, particularly among vulnerable subgroups [5,14,15]. At the same time, current intakes of plant-based foods are below FBDG recommendations [9,11]. Ensuring a safe transition to more sustainable diets requires a more holistic approach by increasing nutrient-dense plant-based foods and moderately reducing animal-sourced foods. Increased plant-based foods, reduced GHGE, and improved diet quality mark progress, but further changes are needed to fully align with sustainable diet targets, highlighting the challenge and importance of a whole-diet approach.

Although sustainability is not incorporated into FBDGs on the island of Ireland, FBDGs of several other countries, such as Canada, Germany, and Denmark, do consider environmental impacts [[31], [32], [33]]. These guidelines encourage intakes from a variety of protein sources and an increase in other plant-based foods such as fruit, vegetables, and whole grains [[31], [32], [33]]. MyPlanetDiet findings show that when appropriately supported, people are willing to eat a variety of protein rich foods, including different meats, fish, and plant-based proteins. Individuals who received personalized feedback for a more sustainable diet increased their intakes of legumes, nuts, or seeds, soy or meat alternative products, and fish while decreasing intakes of red and other meats. Both the control and intervention feedback reduced meat intakes, but the intervention group were consuming less meat at end point. Although the feedback differed in messaging and serving size guidance, the intervention’s lower meat recommendations aligned more closely with what is considered a sustainable healthy diet. This suggests that considering habitual diets and cultural factors like larger meat portions can help tailor dietary advice for specific food groups. Ultimately, the results of the MyPlanetDiet study demonstrate that individuals were willing and able to manage multiple dietary changes congruently during the study. Furthermore, reported food group intakes in both control and intervention groups at the end of the study were close to traditional FBDGs, demonstrating that, if supported, individuals are able and willing to follow dietary guidelines. The MyPlanetDiet RCT builds on existing recommendations and evidence to provide a new perspective of the feasibility and impacts of dietary advice for sustainable healthy dietary guidelines.

Several large cohort studies report associations between sustainable diets and better health outcomes [34,35]. In a recent study, higher adherence to the Eat-Lancet Commission’s Planetary Health Diet was associated with lower all-cause mortality in a 34-year follow-up [34]. Although more sustainable diets have been linked with better long-term health, there is no causal evidence that following sustainable diets or reducing diet-related GHGE will improve health outcomes. In this study, the control and intervention groups received dietary advice aligned with FBDGs, which we expected to lead to improvements in metabolic health markers. However, no changes were observed over time in either group. That said, evidence suggests that following specific components of sustainable diets can lead to improved or stable health status [[36], [37], [38]]. Päivärinta et al. [36] tested 3 animal protein–to–plant protein ratios in a 12-wk RCT, finding that higher plant protein improved total cholesterol and LDL cholesterol . Similarly, Crimarco et al. [39] showed lower LDL cholesterol after 8 wks f consuming plant-based meat alternatives. Although plant-based diets show promise, a narrow focus on 1 food group may increase risk of micronutrient inadequacies [14,33,36]. In a dietary advice-based intervention, participants who reduced fat intakes lost weight and reduced LDL cholesterol and triglycerides over 12 mo [37]. Although fat intake decreased in MyPlanetDiet, the shorter duration may explain the lack of metabolic response (12 wk compared with 12 mo) [37]. Additionally, the heterogeneity of this study’s sample may explain variation in metabolic health outcomes, unlike studies targeting more uniform or at-risk populations [[37], [38], [39]]. Future research could examine how at-risk subgroups respond to sustainable dietary advice.

### Strengths and limitations

To our knowledge, this is the first dietary intervention study to test a whole-diet approach to achieving a more sustainable diet. Similarly, we are not aware of any work that has offered personalized nutrition feedback to reduce diet-related GHGE. Our findings offer new insights into transitioning to a more sustainable diet and fill a critical literature gap. The MyPlanetDiet study was conducted using a single-blinded parallel RCT approach with a large sample size (*n* = 355) from 3 sites on the island of Ireland. Researchers used SOPs and underwent centralized training to ensure personalized feedback and intervention management was cohesive across the study sites. To encourage behavior change, participants received personalized feedback through a 1-to-1 diet counseling session with a research dietitian/nutritionist and had regular follow-up e-mails or calls to discuss the study diets. There was a relatively low dropout rate (18%). possibly attributed to the regular communication and support from study dietitians/nutritionists.

As for limitations, per study inclusion criteria, we recruited those consuming moderate-to-high GHG-emitting diets, based on limited criteria (self-reported red meat intake), as we expected to see greater improvements in environmental metrics using moderate-to-high GHG emitters relative to the general population. The environmental metrics used in this study have been published in previous research in the United Kingdom [27]. Data from the United Kingdom was used due to the lack of available data on the island of Ireland, which is an inherent limitation of the work. Most of the outcomes presented in this analysis rely on self-reported dietary data. Dietary intake methods are notoriously challenging, and individuals may report intakes incorrectly. However, our dietary assessment method, Foodbook24, has been previously validated [24]. It may be possible that participants chose to engage in other healthy lifestyle behaviors, such as increased physical activity, during the study; however, we did not measure health and lifestyle behaviors at both time points and cannot account for this in our analysis. Other limitations include that it was not possible to double blind the study as researchers had to generate personalized feedback and provide the diet counseling to participants at baseline. However, most of the findings presented in this study are reported directly from participants who were blinded to their study group. In regard to the MyPlanetDiet study cohort, participants were largely White, living in an urban setting, and highly educated. As interpersonal factors will influence dietary change and physiologic responses to such change, it will be important to test similar dietary recommendations with a more diverse population. The MyPlanetDiet sample population were high meat eaters at baseline and consequently decreased meat intake and increased intakes of legumes during the study. However, if this dietary advice were tested among individuals with different habitual dietary patterns, socioeconomic statuses, ethnicities, or age groups, the magnitude of change might differ, and additional barriers to change could emerge depending on the specific circumstances. Therefore, future work would warrant updated feedback messages and dietary recommendations specific to the target population.

### Conclusion

It is clear that the environmental impact of food systems must be reduced to mitigate the effects of climate change and adhere to global climate targets [1,2]. Meaningful change will require a full-system approach, which means all actors, including consumers, doing their part for a more sustainable food system. Moving individuals toward more sustainable diets is critical, and our findings address an evidence gap to support this transition. We have shown that it is possible at the consumer level to adopt more sustainable diets. Changes to dietary intakes resulted in decreased diet-related GHGE without compromising health status. Further research is warranted in other populations, for longer intervention periods, or with other outcomes measures (ie, nutrient status). However, our findings add to the literature for what the transition to sustainable diets will look like in the real world. It is important for future FBDGs to consider how to appropriately support individuals to make dietary changes. Our findings show that supporting individuals with personalized nutrition led to dietary change for more sustainable and healthy diets without negative health consequences.

## Author contributions

The authors’ responsibilities were as follows—KPD, ERG, AMO: performed statistical analysis and wrote the paper; ERG, MEK, JVW, APN, SNM, AMO; designed the research; KPD, UML, LL, MCC: conducted the research; AMO: had primary responsibility for final content; and all authors: have read and approved of the final manuscript.

## Data availability

Data described in the manuscript, code book, and analytic code will be made available upon request pending corresponding author approval, ethical approval, and data management agreements.

## Funding

The SuHe Guide project is funded through the Department of Agriculture, Food, the Marine (DAFM) (info@agriculture.gov.ie)/Food Institutional Research Measure (FIRM) (grant number 2019R546), and Department of Agriculture, Environment and Rural Affairs (DAERA) (daera.helpline@daera-ni.gov.uk) (grant number 19/R/546). DAFM and DAERA had no role in the design, analysis or writing of this article.

## Conflicts of interest

The authors report no conflicts of interests.
